# Feasibility of targeted cascade genetic testing in the family members of BRCA1/2 gene pathogenic variant/likely pathogenic variant carriers

**DOI:** 10.1038/s41598-022-05931-3

**Published:** 2022-02-03

**Authors:** Jeeyeon Lee, Ji Yeon Ham, Ho Yong Park, Jin Hyang Jung, Wan Wook Kim, Byeongju Kang, Yee Soo Chae, Soo Jung Lee, In Hee Lee, Nan Young Lee

**Affiliations:** 1 Department of Surgery, School of Medicine, Kyungpook National University, Daegu, Republic of Korea; 2 Department of Clinical Pathology, School of Medicine, Kyungpook National University, Daegu, Republic of Korea; 3 Department of Hemato/Oncology, School of Medicine, Kyungpook National University, Daegu, Republic of Korea; 4 Kyungpook National University Chilgok Hospital, Daegu, Republic of Korea

**Keywords:** Cancer, Genetics

## Abstract

The pathogenic variant (PV) or likely pathogenic variant (LPV) *BRCA1/2* gene is strongly associated with hereditary breast or ovarian cancer. Therefore, it is important to screen blood relatives to establish preventive modalities and surveillance. This study evaluated the feasibility of targeted cascade genetic testing for family members of *BRCA1/2* gene PV or LPV carriers. We screened 18 families for *BRCA1/2* gene status via the conventional cascade genetic test (n = 9) and targeted cascade genetic test (n = 9), which targeted the exon region wherein the index patient showed PV or LPV. The pedigree and clinicopathologic characteristics were reviewed and analyzed. All index patients were diagnosed with breast cancer, while the third family members were all healthy. In the conventional cascade test group, 3 index patients and 3 family members had the *BRCA1/2* gene PV or LPV. In the targeted cascade test group, 5 family members had same type of *BRCA1/2* gene PV or LPV as their index patients. Two families had an identical string of *BRCA1/2* gene PV or LPV. Although the targeted cascade genetic test cannot completely characterize the *BRCA1/2* gene, it is sufficient for determining its PV or LPV status. This limited genetic test can be used for family members of PV or LPV carriers.

## Introduction

*BRCA1/2* genes are originally tumor suppressor genes which have a critical role in human DNA repair. These are highly associated with hereditary breast or ovarian cancer when these genes are of the pathogenic variant (PV) or likely pathogenic variant (LPV)^1–3^. The cumulative risk of breast and ovarian cancer for females with the *BRCA1/2* gene PV or LPV increases with age, specifically with an estimated penetrance of 60–70% and 20–30% for breast and ovarian cancer, respectively, by the age of 70^4–6^. Therefore, risk-reducing salpingo-oophorectomy (RRSO) or bilateral prophylactic mastectomy can be done to prevent breast or ovarian cancer in *BRCA1/2* gene carriers^7,8^.

Because the inheritance of the *BRCA1/2* gene follows an autosomal dominant pattern, the affected gene has a 50% chance of being inherited to biological children regardless of sex and the exon with PV or LPV can be inherited only in the same type^9,10^. Cascade testing involves genetic evaluation of the blood relatives of individuals carrying specific genetic mutations^11,12^. The first individual affected with *BRCA1/2* gene PV or LPV in a family is defined as the index patient or proband, while their first- and second-degree blood relatives should undergo genetic counseling and cascade genetic testing for *BRCA1/2* gene.

Theoretically, family members and their index patient should have identical PV or LPV of the *BRCA1/2* gene. Therefore, it may be possible to use the targeted cascade genetic test, wherein the index patient tests only a specific exon showing a PV or LPV in the gene of a family member. This method is more time- and cost-efficient than conventional testing. This study analyzed the results of the *BRCA1/2* gene in conventional and targeted cascade genetic testing to evaluate the feasibility of a targeted cascade genetic test for *BRCA1/2* gene.

## Results

Among the 9 families, a total of 19 and 21 individuals underwent conventional and targeted cascade genetic test for the *BRCA1/2* gene, respectively. In the targeted cascade test group, 3 male individuals were included. All index patients were diagnosed with breast cancer, while 2 of these patients the in conventional cascade test group had a concomitant diagnosis of ovarian cancer. In both groups, the third family members (family member #3) were all healthy. In family #1 of the conventional cascade test group, 3 index patients and 3 family members had *BRCA1/2* gene PV or LPV. On the other hand, 5 family members (all were family member #1) in the targeted cascade test group had the same type of PV or LPV *BRCA1/2* gene as their index patients (Table 1).

**Table 1:** Demographics of 18 family groups who underwent conventional and targeted cascade testing for *BRCA1/2* genes.

	Conventional cascade test	Targeted cascade test
No. of family groups (people)	9 (19)	9 (21)
Gender (M:F)	0: 19	3: 18
**No. of family members**		
Index patients	9	9
Family member #1	9	9
Family member #2	1	3
Underlying malignancy*		
Index patient	Breast cancer (n = 9) Ovarian cancer (n = 2)	Breast cancer (n = 9)
Family member #1	Breast cancer (n = 9)	Healthy (n = 9)
Family member #2	Healthy (n = 1)	Healthy (n = 3)
***BRCA1/2* gene status**		
Index patient	PV/LPV^†^ (n = 3) VUS^‡^ (n = 3) Wild type (n = 3)	PV/LPV (n = 9)
Family member #1	PV/LPV (n = 2) VUS (n = 3) Wild type (n = 4)	Detected PV/LPV (n = 5) Not detected PV/LPV (n = 4)
Family member #2	PV/LPV (n = 0) VUS (n = 1) Wild type (n = 0)	Detected PV/LPV (n = 0) Not detected PV/LPV (n = 3)

Underlying diseases could be duplicated; ^†^*PV/LPV* pathogenic variant/likely pathogenic variant; ^‡^*VUS* variant of uncertain significance.

**Figure 1:**
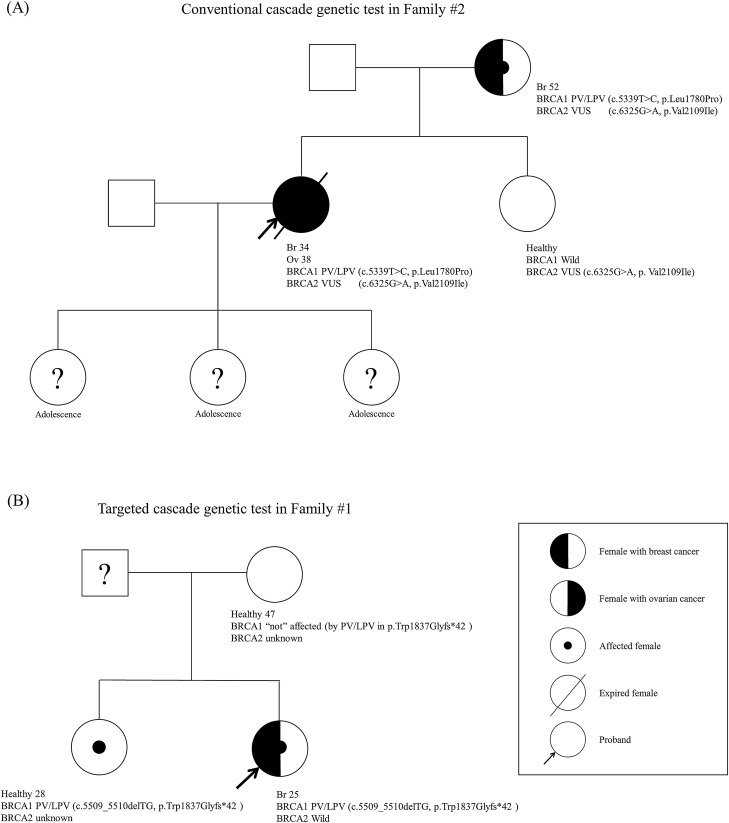
Schematic pedigree of families who underwent cascade genetic tests for *BRCA1/2* genes. (**A**) Pedigree of family #2 who underwent conventional cascade genetic testing for *BRCA1/2* genes. (**B**) Pedigree of family #1 who underwent targeted cascade genetic test for *BRCA1/2* genes.

In family #2 of the conventional test group, the *BRCA1* gene PV or LPV was identified in 2 family members, while *BRCA2* gene variants of uncertain significance (VUS) were identified in 3 family members (Fig. 1A). In family #4 of the conventional test group, 2 family members had exactly the same results of *BRCA1/2* gene. However, the other 7 families had varying results of *BRCA1/2* gene among the family members (Table 2).

**Table 2:** Results of conventional cascade genetic testing for gBRCA1/2 status.

No. Group #	Gene	Index patient		Family member #1		Family member #2	
		Status	Variants	Status	Variants	Status	Variants
1	BRCA1	Wild	–	Wild			
	BRCA2	VUS^†^	c.8415G>T, p.Lys2729Asn	Wild			
2	BRCA1	PV^‡^	c.5339T>C, p.Leu1780Pro	PV	c.5339T>C, p.Leu1780Pro	Wild	–
	BRCA2	VUS	c.6325G>A, p.Val2109Ile	VUS	c.6325G>A, p.Val2109Ile	VUS	c.6325G>A, p.Val2109Ile
3	BRCA1	PV	c.5080G>T, p.Glu1694*	PV	c.5080G>T, p.Glu1694*		
	BRCA2	Wild	–	Wild	–		
4	BRCA1	Wild	–	Wild	–		
	BRCA2	VUS	c.7976 + 45G>C, -	Wild	–		
5	BRCA1	VUS	c.7976 + 24G>A, -	Wild	–		
	BRCA2	Wild	–	Wild	–		
6	BRCA1	Wild	–	Wild	–		
	BRCA2	VUS	c.572A>T, pAsp191Val	Wild	–		
7	BRCA1	Wild	–	Wild	–		
	BRCA2	Wild	–	Wild	–		
8	BRCA1	Wild	–	Wild	–		
	BRCA2	VUS	c.7522G>A, p.Gly2508Ser	Wild	–		
9	BRCA1	PV	c.1831del, p.Leu611*	Wild	–		
	BRCA2	Wild		VUS	c.3132T>G, p.Cys1044Trp		

^†^*VUS* variant of uncertain significance, ^‡^*PV* pathogenic variant.

The targeted cascade genetic test group consisted of the index patients with breast cancer (n = 9) and healthy family members: family member #2 (n = 9) and #3 (n = 3). Among the 9 families, 5 of those designated as family member #2 had an identical *BRCA1/2* gene PV or LPV status as their respective index patient (Table 3). In family #1 of the targeted test group, the *BRCA1* gene PV or LPV was identified in their sibling, but not in the mother. *BRCA2* gene status was only evaluated in the index patient (Fig. 1B).

**Table 3:** Results of targeted cascade genetic testing for gBRCA1/2 status.

No. Group	Gene	Index patient		Family member #1		Family member #2	
		Status	Variants	Status	Variants	Status	Variants
1	BRCA1	PV^†^	c.5509_5510delTG, p.Trp1837Glyfs*42	PV	c.5509_5510delTG, p.Trp1837Glyfs*42	Wild	–
	BRCA2	Wild	–				
2	BRCA1	PV	c.5080G>T, p.Glu1694*	PV	c.5080G>T, p.Glu1694*	Wild	–
	BRCA2	Wild	–				
3	BRCA1	LPV^‡^	c.4902_4903delGGinsCT, p.Arg1634_Glu1635delinsS*	Wild	–	Wild	–
	BRCA2	Wild	-				
4	BRCA1	PV	c.1831del, p.Leu611*	Wild	–		
	BRCA2	Wild	–				
5	BRCA1	Wild	–				
	BRCA2	PV	c.5576_5579delTTAA, p.Ile1859Lysfs*3	Wild	–		
6	BRCA1	PV	c.922_924delinsT, p.Ser308*	PV	c.922_924delinsT, p.Ser308*		
	BRCA2	Wild	–				
7	BRCA1	PV	c.5339T>C, p.Leu1780Pro	Wild			
	BRCA2	Wild	–				
8	BRCA1	Wild	–				
	BRCA2	PV	c.994delA, p.Ile332fs	PV	c.994delA, p.Ile332fs		
9	BRCA1	Wild	–				
	BRCA2	PV	c.5576_5579delTTAA, p.Ile1859fs	PV	c.5576_5579delTTAA, p.Ile1859fs		

^†^*PV* pathogenic variant, ^‡^*LPV* likely pathogenic variant.

There were reasons for genetic evaluation other than a family history of breast cancer, such as young age, bilateral breast cancer, and personal history of breast cancer (Supplementary Table 1). All index patients (i.e., those diagnosed with breast cancer) and those designated as family member #2 in the conventional cascade genetic test group have their clinicopathologic factors described in Supplementary Table 2. Moreover, 2 index patients and 1 family member #2 had undergone RRSO, while another family member #2 had also underwent contralateral prophylactic mastectomy.

## Discussion

There are several methods for the evaluation of *BRCA1/2* gene, such as direct (Sanger) DNA sequencing, quantitative polymerase chain reaction (PCR)-based techniques, multiplex ligation-dependent probe amplification, and next-generation sequencing (NGS) techniques^15^. Even if the direct (Sanger) DNA sequencing is considered the gold standard method and is relatively low-cost for *BRCA1/2* gene evaluation, this method cannot detect large genomic rearrangements (LGRs). On the other hand, quantitative PCR can detect LGRs, but it is a labor-intensive technique. To overcome these drawbacks, NGS technology is used; this has many advantages including high throughput, lower cost, automated analysis, and use of less amount of DNA^16–19^. However, since it requires a higher start-up cost, complex workflows, dedicated data storage and the turnaround time is quite long (2–4 weeks)^20,21^. This process may delay the decision for simultaneous RRSO or contralateral prophylactic mastectomy with cancer surgery in *BRCA1/2* gene PV or LPV carriers with breast cancer.

Cascade genetic testing refers to genetic counseling and testing in blood relatives of individuals with specific​ genetic mutations^11,12^. Theoretically, the inherited PV or LPV should be identical between proband and blood relatives, if identified, within a biological family. Therefore, the *BRCA1/2* gene of family members can be selectively conducted for the targeted exon region, which can reveal *BRCA1/2* gene PV or LPV in probands. This is referred to as a targeted cascade genetic test, and it can greatly save in cost and interpretation time. We conducted conventional and targeted cascade genetic testing in 18 families. In the conventional test group (n = 9), we found 3 families in which the proband had identical *BRCA1/2* gene PV or LPV results. In the targeted test group (n = 9), we also found 5 families in which the proband had identical *BRCA1/2* gene PV or LPV results in the targeted exon, with no new type of PV or LPV other than that of the proband.

Screening for *BRCA1/2* gene in the general population is practically difficult in time and economic aspect. The cascade genetic test is a very efficient screening test for probands with *BRCA1/2* gene PV or LPV. However, the targeted cascade genetic test is more effective in identifying whether the targeted PV or LPV exists in specific family members. This would save a lot of time in interpretation by skipping the clinically insignificant benign variants.

Unfortunately, when the targeted cascade genetic test is performed, information regarding other exons cannot be obtained, even if there is a potential risk of *BRCA1/2* gene VUS. This can be a problem because several *BRCA1/2* gene VUS have been re-classified as PV or LPV according to their prevalence in specific racial/ethnic groups^22–25^. Without information about other exons, the complete prevention of breast or ovarian cancer cannot be achieved, even if a specific VUS has been re-classified as PV or LPV. However, if another *BRCA1/2* gene VUS or PV or LPV different from that of index patient is found later in the blood relative, the targeted cascade genetic test can be repeated.

Although the targeted cascade genetic test cannot completely characterize the *BRCA1/2* gene in its entirety, it can nevertheless serve as an effective screening modality for healthy blood relatives of PV or LPV carriers. It is practically impossible to perform *BRCA1/2* gene screening for the general population, and the evaluation of whole *BRCA1/2* gene may be burdensome to healthy blood relatives in economic and time aspect. Whereas, the targeted cascade genetic test is a more efficient, cost-effective, and time-saving modality, because it is conducted in selected nucleotide sequences showing the PV or LPV of the index patient.

## Methods

Between January 2014 and June 2021, a total of 1,274 patients with breast cancer had undergone genetic assessments to evaluate *BRCA1/2* gene status at Kyungpook National University Chilgok Hospital, Daegu, Republic of Korea. *BRCA1/2* gene screening was conducted based on the following criteria: diagnosis of breast cancer at < 40 years of age, bilateral breast cancer, personal or family history of breast or ovarian cancer in first- or second-degree relatives, and male breast cancer. Before conducting genetic assessment, all patients received genetic counseling from highly qualified genetic experts and provided written informed consent.

**Figure 2:**
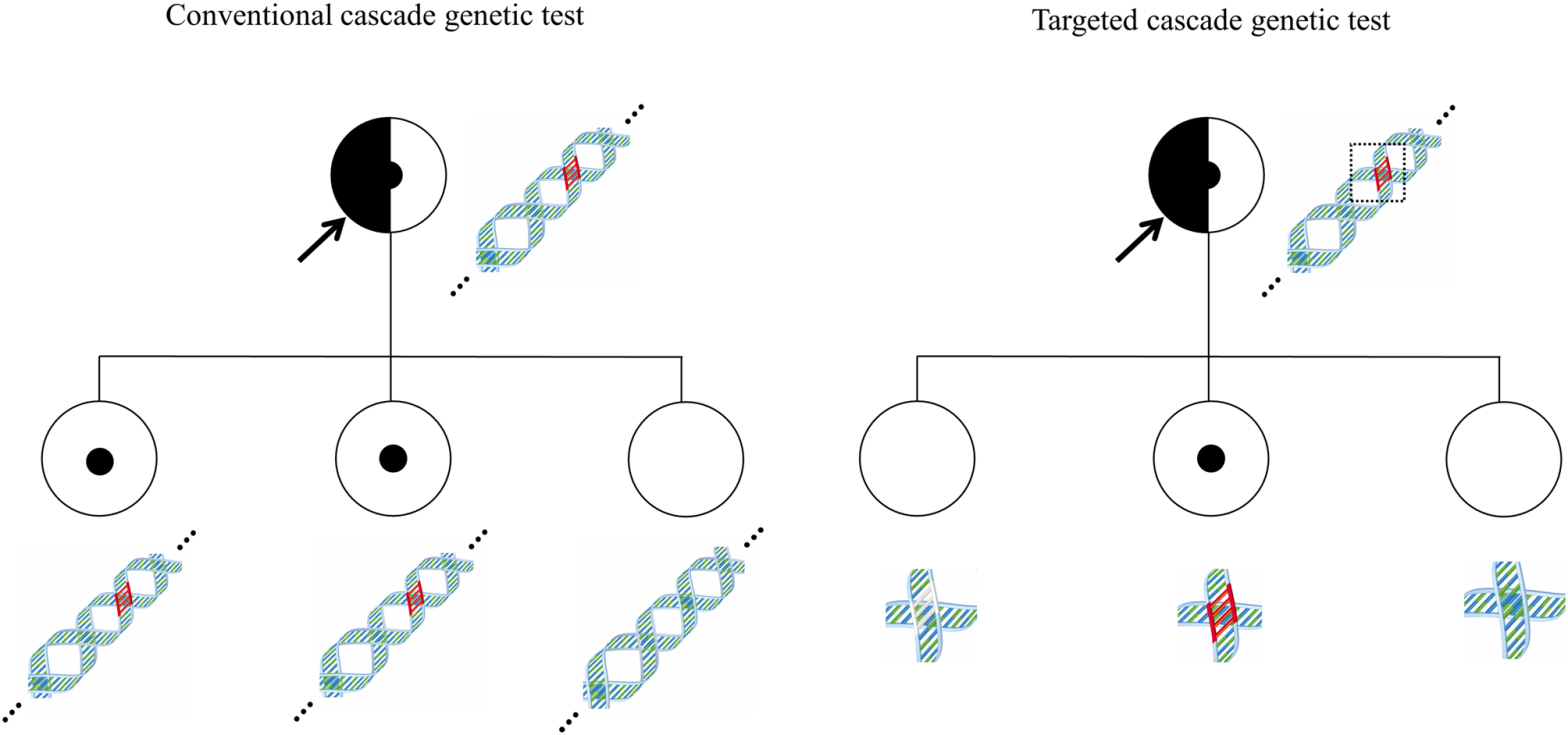
Definition of conventional and targeted genetic cascade test for *BRCA1/2* genes. The conventional cascade genetic testing is defined as testing the relevant genes for blood relatives, whereas targeted cascade testing for *BRCA1/2* gene was defined as testing only a part of exon with PV or LPV of blood relatives of the index patient with *BRCA1/2* gene PV or LPV.

Cascade genetic testing is defined as testing the relevant genes for blood relatives according to the NCI's dictionary of genetic terms; this is referred to as the conventional cascade test in this study. Meanwhile, targeted cascade testing for *BRCA1/2* gene was defined as testing only a part of exon with PV or LPV to blood relatives of the index patient with *BRCA1/2* gene PV or LPV (Fig. 2).

Genomic DNA was extracted from EDTA-treated whole blood by using the Chemagic™ Magnetic Separation Module I (MSM I) method (PerkinElmer chemagen, Baesweiler, Germany) with the DNA Blood 200 μl Kit and QIAamp^®^ DSP DNA Mini kit (QIAGEN GmbH, Hilden, Germany).

The *BRCA1/2* gene screening test and conventional cascade test were conducted by two methods. One was PCR with a direct sequencing method as previously described^13^ and the other was the NGS method. The PCR was conducted using the SimpliAmp thermal cycler (Applied BioSystems, Foster City, CA, USA) with the QIAGEN^®^ Multiplex PCR *plus* kit (QIAGEN) and *Accupower*^®^ PCR PreMix (Bioneer Corp., Daejeon, Republic of Korea). The direct sequencing method based on Sanger sequencing was conducted using the 3500xL Dx Genetic Analyzer (Applied BioSystems). From January 2014 to October 2019, the NGS was performed using the Celemics Library Prep Kit (Celemics Co., Ltd., Seoul, Republic of Korea) and Illumina MiseqDX platform (Illumina Co., Ltd., San Diego, CA, USA). The DNA sequence reads were aligned to reference sequences based on the public human genome build GRCh37/UCSC hg19. Alignment was done with the BWA-mem (version 0.7.10), and variant calling was performed with HaplotypeCaller of Genome Analysis Tool kit (GATK, version 3.5) (Broad Institute, Cambridge, MA, USA). From November 2019, the NGS was performed using the BRCAaccuTest™ PLUS (NGeneBio, Seoul, Republic of Korea) and Illumina MiseqDX platform. The BRCAaccuTest pipeline (version 1.5.0) (NGeneBio) was used for the bioinformatics analysis. The basic concepts of clinical interpretation of detected mutations were in accordance with the 2015 ACMG/AMP guidelines^14^. The results of the *BRCA1/2* gene analysis were reported as wild type (no pathogenic variants), VUS, or PV and LPV; all benign variants were not reported by laboratory physicians (Supplementary Fig. 1).

The targeted cascade test for *BRCA1/2* gene was also conducted via PCR with a direct sequencing method mentioned above for specific target exons with PV or LPV.

This study included 18 families with family history of breast cancer. *BRCA1/2* gene status and clinicopathologic characteristics of breast cancer in all family members were accessible. A total of 9 families underwent conventional cascade genetic test for *BRCA1/2* gene, while the other 9 families underwent targeted cascade genetic test for the exact site where the DNA sequence had changed (i.e., either deletion, frameshift, missense, or nonsense mutation). The protocol used in this study was approved by the Institutional Review Board Committee of the Kyungpook National University Chilgok Hospital (KNUCH 2020-02-026).

The clinicopathologic characteristics and *BRCA1/2* gene status of patients and their family members were reviewed and analyzed. The pedigree was designed using the standard symbols.

### Ethical approval

All procedures performed in studies involving human participants were in accordance with the ethical standards of the institutional and/or national research committee and with the 1964 Helsinki declaration and its later amendments or comparable ethical standards. Ethical approval for the study was obtained from the Institutional Review Board of the KNUCH (KNUCH KNUCH 2020-02-026).

### Informed consent

Informed consent was obtained from all individual participants included in the study.

## Supplementary Information

Supplementary Information 1.

Supplementary Information 2.

Supplementary Information 3.

## Data Availability

The data sets generated and/or analyzed in this study are not publicly available. However, this can be obtained from the corresponding author upon reasonable request.
