# Supplementary figures and images for: Feasibility of targeted cascade genetic testing in the family members of BRCA1/2 gene pathogenic variant/likely pathogenic variant carriers
Source: Sci Rep. 2022 Feb 3;12:1842. doi: 10.1038/s41598-022-05931-3 (PMC8813990; doi:10.1038/s41598-022-05931-3)

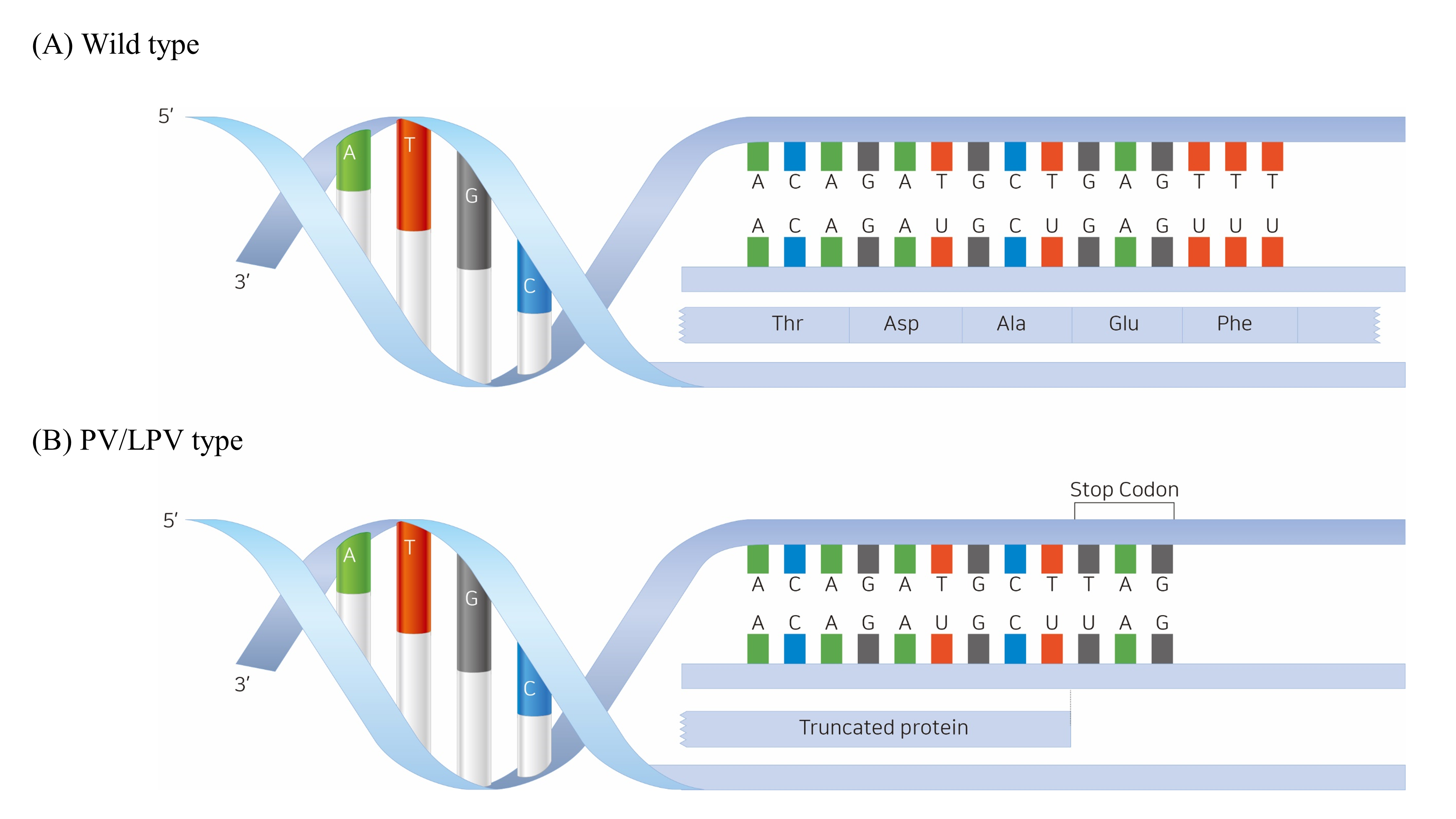

Supplement: Supplementary file 1 — Supplementary Information 1. [file 41598_2022_5931_MOESM1_ESM.tif]
