# Supplementary material for: Feasibility of targeted cascade genetic testing in the family members of BRCA1/2 gene pathogenic variant/likely pathogenic variant carriers
Source: Sci Rep. 2022 Feb 3;12:1842. doi: 10.1038/s41598-022-05931-3 (PMC8813990; doi:10.1038/s41598-022-05931-3)
Supplement: Supplementary file 2 — Supplementary Information 2. [file 41598_2022_5931_MOESM2_ESM.docx]

Supplementary Table 1. Demographics of 9 family groups who underwent conventional cascade genetic testing for *BRCA1/2* genes

|  | Index patient  (n = 9) | Family member #1  (n = 9) | Family member #2  (n = 1) |
| --- | --- | --- | --- |
| Age at genetic evaluation (mean ± SD, years) | 53.5 ± 2.4 | 52.8 ± 2.9 | 33 |
| Age at diagnosis of breast cancer (mean ± SD, years) | 52.9 ± 5.7 | 51.6 ± 4.2 | - |
| Underlying disease (n, %) |  |  |  |
| Breast cancer | 9 (100.0) | 9 (100.0) | - |
| Ovarian cancer | 2 (22.2) | - | - |
| Healthy | - | - | 1 (11.1) |
| Reasons for genetic evaluation (n, %) |  |  |  |
| Family history | 9 (100.0) | 9 (100.0) | 1 (11.1) |
| Young age | 2 (22.2) | - | - |
| Bilateral breast cancer | 2 (22.2) | 1 (11.1) | - |
| Personal history of breast or ovarian cancer | 2 (22.2) | - | - |
| Location of breast cancer (n, %) |  |  |  |
| Right | 5 (55.6) | 6 (66.7) | - |
| Left | 3 (33.3) | 3 (33.3) | - |
| Both | 1 (11.1) |  |  |
| *BRCA1/2* genes status (n, %) |  |  |  |
| Pathogenic variant/Likely pathogenic variant | 2 (22.2) | 3 (33.3) | - |
| Variant of Uncertain Significance | 6 (66.7) | - | - |
| Wild type | 1 (11.1) | 6 (66.7) | 1 (11.1) |
| Concordance of *BRCA1/2* genes with index patient (n, %) | N/A | 3 (33.3) | 0 |

Supplementary Table 2. Clinicopathologic of index patients and related family members who underwent conventional cascade genetic testing for *BRCA1/2* genes

|  | Index patient  (n = 9) | Family member #1  (n = 9) |
| --- | --- | --- |
| Surgery for breast cancer (n, %) |  |  |
| Breast conserving surgery | 4 (44.4) | 7 (77.8) |
| Mastectomy | 5 (55.6) | 2 (22.2) |
| Surgery for axillary lymph nodes (n, %) |  |  |
| Sentinel lymph nodes biopsy | 4 (44.4) | 8 (88.9) |
| Axillary lymph nodes dissection | 5 (55.6) | 1 (11.1) |
| Clinical tumor size (mean±SD, cm) | 2.9 ± 1.6 | 2.0 ± 1.4 |
| Pathologic tumor size (mean±SD, cm) | 2.0 ± 1.5 | 1.7 ± 1.4 |
| Axillary lymph nodes metastasis (n, %) | 4 (44.4) | 2 (22.2) |
| Pathologic stage |  |  |
| Stage 0 | 1 (11.1) | - |
| pCR (including DCIS only) | 2 (22.2) | - |
| Stage IA | 2 (22.2) | 6 (66.7) |
| Stage IIA | 1 (11.1) | 2 (22.2) |
| Stage IIB | 1 (11.1) | - |
| Stage IIIA | 1 (11.1) | 1 (11.1) |
| Stage IIIC | 1 (11.1) | - |
| Immunohistochemical staining results (n, %) |  |  |
| Estrogen receptor, positive | 6 (66.7) | 6 (66.7) |
| Progesterone receptor, positive | 4 (44.4) | 6 (66.7) |
| Her2/neu gene, positive | 2 (22.2) | - |
| Triple negative breast cancer | 3 (33.3) | 3 (33.3) |
| High Ki67 index (>15%) | 5 (55.6) | 6 (66.7) |
| Neoadjuvant chemotherapy (n, %) | 4 (44.4) | - |
| Adjuvant chemotherapy (n, %) | 4 (44.4) | 3 (33.3) |
| Adjuvant radiotherapy (n, %) | 8 (88.9) | 7 (77.8) |
| Target therapy (n, %) | 2 (22.2) | 0 |
| Adjuvant endocrine treatment (n, %) | 6 (66.7) | 6 (66.7) |
| Contralateral prophylactic mastectomy (n, %) | 0 | 1 (11.1) |
| Risk-reducing salphigo-oophorectomy (n, %) | 2 (22.2) | 1 (11.1) |
