# Supplementary material for: Feasibility of targeted cascade genetic testing in the family members of BRCA1/2 gene pathogenic variant/likely pathogenic variant carriers
Source: Sci Rep. 2022 Feb 3;12:1842. doi: 10.1038/s41598-022-05931-3 (PMC8813990; doi:10.1038/s41598-022-05931-3)
Supplement: Supplementary file 3 — Supplementary Information 3. [file 41598_2022_5931_MOESM3_ESM.docx]

Supplementary Figure legends

Supplementary Figure 1. Status of *BRCA1/2* genes. (A) Wild type. No pathogenic variant/likely pathogenic variant (PV/LPV) variant is confirmed. (B) PV/LPV type. Because the TAG DNA sequence is a stop codon, the DNA could no longer be translated, and the protein is truncated. This type is strongly associated with hereditary breast or ovarian cancer.
